# Artificial Intelligence Supports Automated Characterization of Differentiated Human Pluripotent Stem Cells

**DOI:** 10.1093/stmcls/sxad049

**Published:** 2023-06-26

**Authors:** Katarzyna Marzec-Schmidt, Nidal Ghosheh, Sören Richard Stahlschmidt, Barbara Küppers-Munther, Jane Synnergren, Benjamin Ulfenborg

**Affiliations:** Department of Soil and Environment, Swedish University of Agricultural Sciences (SLU), Skara, Sweden; Takara Bio Europe, Gothenburg, Sweden; Department of Biology and Bioinformatics, School of Bioscience, University of Skövde, Skövde, Sweden; Department of Biology and Bioinformatics, School of Bioscience, University of Skövde, Skövde, Sweden; Takara Bio Europe, Gothenburg, Sweden; Department of Biology and Bioinformatics, School of Bioscience, University of Skövde, Skövde, Sweden; Department of Molecular and Clinical Medicine, Institute of Medicine, Sahlgrenska Academy at University of Gothenburg, Gothenburg, Sweden; Department of Biology and Bioinformatics, School of Bioscience, University of Skövde, Skövde, Sweden

**Keywords:** pluripotent stem cells, cell differentiation, hepatocytes, quality control, artificial intelligence, image analysis, computer-assisted

## Abstract

Revolutionary advances in AI and deep learning in recent years have resulted in an upsurge of papers exploring applications within the biomedical field. Within stem cell research, promising results have been reported from analyses of microscopy images to, that is, distinguish between pluripotent stem cells and differentiated cell types derived from stem cells. In this work, we investigated the possibility of using a deep learning model to predict the differentiation stage of pluripotent stem cells undergoing differentiation toward hepatocytes, based on morphological features of cell cultures. We were able to achieve close to perfect classification of images from early and late time points during differentiation, and this aligned very well with the experimental validation of cell identity and function. Our results suggest that deep learning models can distinguish between different cell morphologies, and provide alternative means of semi-automated functional characterization of stem cell cultures.

## Graphical Abstract

**Graphical Abstract:**
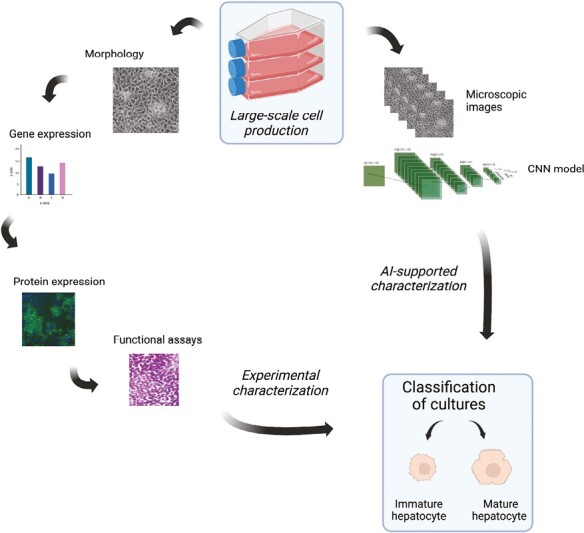

**Significance Statement**

This study demonstrates that a convolution neural network can accurately predict the differentiation stage of stem cells differentiating toward hepatocytes, based on cell morphology in microscopy images. This potentially allows for semi-automated functional characterization of stem cells, to reduce costs, time, and expertise required to experimentally characterize differentiated stem cells.

## Introduction

Developments in artificial intelligence (AI) over the past decade have promoted a surge of applications in different fields of science. Importantly, the success of deep learning has revolutionized image analysis, not only in computer vision but also in medical and cellular imaging. In contrast to traditional machine learning techniques, deep learning networks are able to automatically and efficiently learn higher-level representations of data without the need for manual feature engineering.^1^ Within the field of stem cell research, deep learning applied to cellular images holds the potential for accurate and automated analysis of cell cultures, as recent studies have demonstrated.^2-7^ In the study by Imamura et al., induced pluripotent stem cells (iPSCs) were generated from cells from healthy controls and patients with amyotrophic lateral sclerosis. The iPSCs were differentiated into motor neurons and immunostained, followed by image classification where 90% of the cell images were correctly classified.^4^ Maddah et al. investigated the structural toxicity of iPSC-derived hepatocytes and cardiomyocytes by treating cells with known toxic and non-toxic compounds. A network model trained on fluorescence microscope images was able to correctly identify structural changes from toxic compounds for both cell types.^6^ By developing a new ensemble-based deep learning method, Joy et al. were able to identify individual nuclei in dense human iPSC (hiPSC) colonies and perform cell tracking to study cell behavior over time.^5^

A commonly used deep learning architecture specialized for image classification is the convolutional neural network (CNN). It performs mathematical operations to translate an image of pixels into so-called feature maps, which represent visual features like edges and shapes. Several convolutional layers can be stacked on top of each other, each taking the previous map as input, such that the CNN learns higher-level features that are more informative for image classification.^8^ Several recent studies have demonstrated the feasibility of using CNNs for predicting stem cell differentiation states based on microscopy images. Waisman et al. differentiated mouse embryonic stem cells (mESCs) into epiblast-like cells, and trained a deep learning model to distinguish between culture images of mESCs and differentiated cells. They achieved perfect classification accuracy after only 6 h following the onset of differentiation.^9^ Similarly, Liu et al. differentiated human embryonic stem cells (hESCs) into trophoblast-like cells and trained a model to distinguish between trophoblast morphology and hESCs. Two networks used achieved >99 % accuracy on day 12.^10^ In studies by Zhu et al.^11^ and Lan et al.,^12^ single-cell images were used instead to successfully recognize neural and osteogenic differentiation, respectively.

The immense expectations on pluripotent stem cells (PSCs) in many areas of biomedical research place high demands on production procedures and rigorous quality control of stem cell cultures, that is, to identify successfully differentiated cultures, and to verify cell marker expression, cellular morphology, and functionality. Since this is routinely done experimentally with microscopic inspections, qPCR, immunocytochemistry, and various functional assays, the process is costly, time-consuming, and requires highly trained specialists. Hence there is a need for more efficient validation methods of PSC identity and function.^13^ If these steps could be automated through deep learning-based image analysis, large reductions in cost, and workload required for stem cell production could be made.

A few studies have explored the potential of using deep learning for quality control and automated assessment of stem cell cultures. Orita et al. trained a CNN to distinguish between hiPSC-derived cardiomyocyte cultures’ based on whether they were judged suitable for downstream experiments.^14^ The model achieved close to 90% accuracy, demonstrating the possibility of using deep learning for automated hiPSC quality control. In a study by Hirose et al., a deep learning method was developed for automated cell tracking of keratinocyte stem cells.^15^ The aim was to enable non-invasive and efficient quality control of cell cultures proliferative capacity, as an alternative to single-cell clonal analysis, which is expensive and demanding to perform. By relying on cell motion data from the deep learning method, the authors were able to significantly increase the probability of obtaining proliferative stem cell colonies. Similarly, Piotrowski et al. developed a deep learning method for non-invasive automated cell state recognition of hiPSC colonies.^16^ This method was able to distinguish between different cell states, such as hiPSC colony, differentiated cells, and dead cells, more accurately than a human expert.

The aim of the present study is to provide a proof-of-concept that CNNs can be used for automated quality control of cultures undergoing hepatocyte differentiation, as a complement to visual inspection of cultures by an expert, to evaluate the maturity of the stem cell-derived hepatocyte cultures. In this study, we hypothesize that the morphological features of cells captured in phase-contrast microscopy images are sufficient to distinguish between early and late stages of hepatocyte differentiation. Late stage corresponds to mature hepatocytes with functional properties that recapitulate many features of primary human hepatocytes.^17,18^ Once a CNN has been trained to recognize these features, it could provide predictions of differentiation stages in line with data obtained from experimental verification.

## Materials and Methods

### Image Data

Convolutional neural networks were trained on phase-contrast microscope photographs of stem cell cultures captured at different time points during differentiation toward hPSC-HEP. Images of hPSCs differentiating into hepatocytes were obtained from Takara Bio Europe AB during routine differentiation processes, applying their current hepatocyte differentiation protocol (Cellartis iPS Cell to Hepatocyte Differentiation System, Cat. No. Y30055, Takara Bio Europe AB; see also section on Human Pluripotent Stem Cells Differentiation below), as well as a previous hepatocyte differentiation protocol also developed by Takara Bio Europe AB.^19^

A total of 1331 images were collected from routine inspections of differentiation batches during a time period of several years. The images were obtained using a phase-contrast microscope (magnification 10×) (EclipseTi-U, Nikon, Amsterdam, The Netherlands), with an ANDOR Zyla sCMOS digital camera and then processed using NIS-Elements software package (version 4.30). Images were labeled as early differentiation (ED, days 1-14, in total of 693 images) and late differentiation (LD, days 16-23, in total of 638 images), respectively. The distinction between ED and LD was based on the change from differentiation to hepatocyte maturation culture medium on day 14.

### Pre-Processing and Augmentation

The dimensions of the original images were between 1280 × 1024 and 2560 × 2160 pixels. To generate a larger number of images for the analysis, each image was cut into a maximum of six patches (1000 × 1000 pixels) using MATLAB. This was carried out for 70% (932), 20% (266), and 10% (133) of the original images separately, and the 3 sets of patches were later used for training, validation, and testing, respectively. The total number of patches generated was 6972. Pixel values for all patches were scaled to values between 0 and 1.

**Figure 1.**
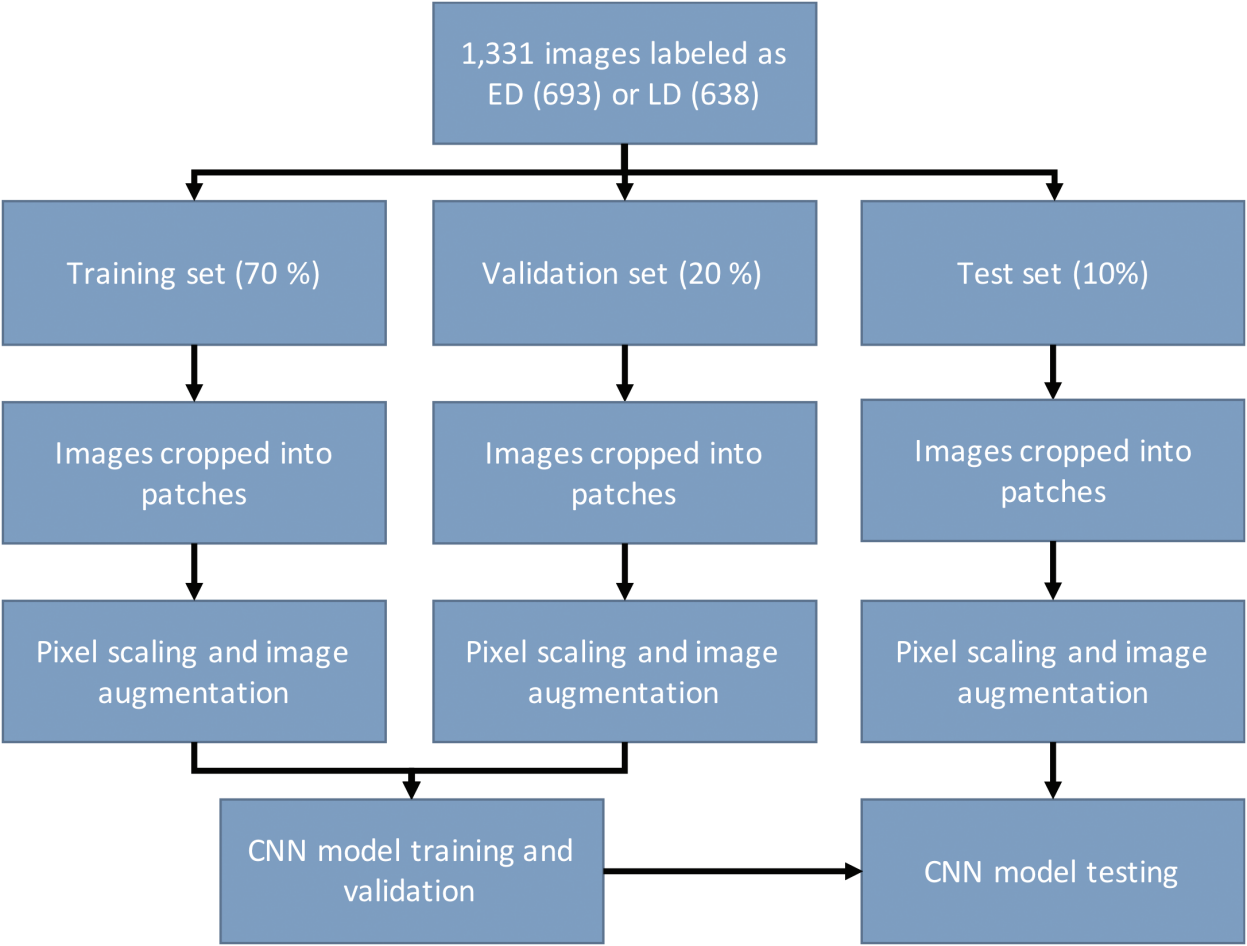
Image processing and analysis workflow. Abbreviations: ED, early differentiation, CNN, convolutional neural network; LD, late differentiation.

Data augmentation was performed and the patches were downsized to 400 × 400, 200 × 200, and 100 × 100 pixels to investigate how image size affects the performance of the CNN. The patches were subsequently randomly flipped (vertically and horizontally) and rotated in the range between −0.2 and +0.2 of 360° rotation with TensorFlow. This step was performed when the patches were given as input to the CNN. The flipped/rotated sets of patches are referred to as LargePatchAug, MediumPatchAug, and SmallPatchAug, respectively, and each set contains 6972 patches. These sets were used to evaluate how data augmentation affects CNN performance. The image processing and analysis workflow are illustrated in Fig. 1.

### Model Specification

**Figure 2.**
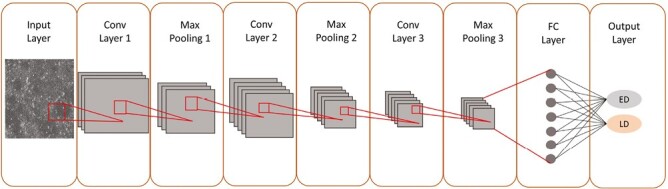
Architecture of the convolutional neural network used in this study. The network consists of 3 convolutional layers extracting features from an input image, 3 max pooling layers reducing the feature map size followed by 2 fully connected layers for image classification. The kernel, a filter extracting features from the image, is represented by a red color square. Batch normalization layers were not applied.

The CNN model was implemented in Python version 3.8.5 in the Anaconda 3 environment using Keras^20^ with TensorFlow 2.3.1 as a backend. The model architecture is illustrated in Fig. 2 and consists of an input layer followed by 3 sets of convolutional/ReLU/max-pooling layers. The last max-pooling layer is connected to a feed-forward network with 1 hidden layer and an output layer with 2 nodes for image classification (ED vs. LD). The output layer used the Softmax activation function so the values of the output nodes sum to 1. This can be regarded as the probability of each image being ED or LD. Images were classified according to the class with the highest probability.

### Training, Validation, and Testing

The He normal initializer was used to initialize the random weights of the CNN. The weights initialized by this method are random but also depend on the size of the upper layer of neurons in the network, allowing a faster and more efficient choice of weight range.^21^ According to Waisman et al.,^9^ Adam, Adamax, and Adagrad optimizers performed equally well when training CNNs for classification of mouse ESCs differentiating into epiblast-like cells. Therefore, Adam, an optimization algorithm designed especially for training deep neural networks,^22^ was used in this study. The loss function used was binary cross entropy since the network was optimized for binary classification of images. The model was fitted for 500 epochs and early stop with a patience of 50 epochs. This allowed the model to stop training earlier if the loss function converges on a minimum value. The training was performed with a learning rate of 0.001 and mini-batch size of 16.

The model was trained on 70% of the LargePatchAug set and 20% was used for validation/hyperparameter optimization. The remaining 10% was reserved for testing. In addition, the following training set sizes were used to assess the impact of the number of training images on model performance: 4096, 3072, 2048, and 1072. The number of images used for validation and testing was constant. Classification performance was reported using accuracy (ACC) and the F1 statistic according to Equations 1 and 2, respectively. True positives (TP) denote LD images classified as LD, false positives (FP) denote ED images classified as LD, true negatives (TN) denote ED images classified as ED, and false negatives (FN) denote LD images classified as ED.


$$\begin{array}{l} ACC=\frac{TP+TN}{TP+FP+TN+FN} \end{array}$$ Equation 1

$$\begin{array}{l} F1=\frac{TP}{TP+\frac{FP+FN}{2}} \end{array}$$ Equation 2

Analyses were performed on a personal computer with Windows 10 Pro 64 bits operating system, 3.3GHz Intel Xenon E-2126G CPU, 32 GB RAM, and NVIDIA Quadro P1000 GPU. GPU parameters were as follows: NVIDIA drivers 430.64, 512 Cuda cores, 4GB of dedicated video memory, and 20 GB of total available graphics memory. The runtime was between 20 min and 2h 15 min, depending on the dataset and hyperparameter settings.

### Human Pluripotent Stem Cells Differentiation

For the experimental validation of CNN predictions, hiPSC lines including Cellartis Human iPS Cell Line 22 (ChiPSC22) (Cat. No. Y00325), Cellartis Human iPS Cell Line 18 (ChiPSC18) (Cat. No. Y00305), and Cellartis Human iPS Cell Line 6b (ChiPSC6b), were obtained from Takara Bio Europe AB (Gothenburg, Sweden). The hiPSC lines were differentiated into human pluripotent stem cell-derived hepatocytes (hiPSC-HEP) by using the Cellartis iPS Cell to Hepatocyte Differentiation System (Cat. No. Y30055, Takara Bio Europe AB) according to the manufacturer’s recommendations.

### RNA Extraction, cDNA Synthesis, and Real Time Quantitative PCR

Cell samples were collected in RNAprotect Cell Reagent (Cat. No. 76526, QIAGEN) during the differentiation procedure on days 9, 11, 14, 16, 18, 21, 23, and 25. RNA was extracted from these samples using MagMAX-96 Total RNA Isolation Kit (Cat. No. AM1830, ThermoFisher). cDNA was synthesized by applying High-Capacity cDNA Reverse Transcription Kit (Cat. No. 4368814, ThermoFisher). Real-time quantitative PCR (RT-qPCR) was performed using TaqMan Fast Advanced Master Mix (Cat. No. 4444557, ThermoFisher) for the following markers: KRT19 (Hs00761767_s1, ThermoFisher), CYP2C9 (Hs04260376_m1, ThermoFisher), MIR370 (Hs04231551_s1, ThermoFisher), FLJ22447(Hs01382450_m1, ThermoFisher), and FLJ22763 (Hs01396927_m1). GAPDH (Hs99999905_m1, ThermoFisher) was used as a reference gene, and cDNA synthesized on a cocktail of RNA extracted from different cell types was used as a calibrator.^19^

### Albumin Secretion Assay

Albumin secretion was determined on days 9, 11, 14, 16, 18, 21, 23, and 25 by applying Albumin Human ELISA Kit (Cat.No. EHALB, ThermoFisher) on 24 h conditioned medium according to the manufacturer’s instruction. The results were normalized to total protein content. Protein content was measured by applying Pierce BCA Protein Assay kit (Cat. No. 23227, ThermoFisher).

### Periodic Acid Schiff Staining

Periodic acid-Schiff Staining (PAS) was performed to detect glycogen storage in mature hiPSC-HEP. Fixed cells were incubated in periodic acid (Cat.No. 3951, Sigma-Aldrich) for 15 min and washed 3 times with dH_2_O. Then the cells were incubated in SCHIFF reagent (Cat. No. 3952016, Sigma-Aldrich) for 30 min. The cells were washed again 3 times with dH_2_O, and incubated in hematoxylin (Cat. No. GHS316, Sigma-Aldrich) for 90 s. Finally, the cells were washed 3 times with dH_2_O.

### Immunocytochemistry

Cells were fixed at days 9, 11, 14, 16, 18, 21, 23, and 25 by incubation in 4% formaldehyde (Cat. No. 02176, Histolab) for 10 min at RT. The cells were permeabilized by incubation in 1% Triton X-100 (Cat.No. T8787, Sigma-Aldrich) in D-PBS +/+ (Cat. No. 14040-091, Gibco) for 15 min at RT, then the cells were blocked in 2% BSA (Cat. No. A9418, Sigma-Aldrich) in D-PBS +/+ for 60 min at RT. The cells were immunostained for the markers CK18 (Cat. No. MA5-12104, Invitrogen) and CAR (Cat. No. MA5-29208, Invitrogen). The antibodies were diluted in 0.1% BSA in D-PBS +/+ (1:100 for primary antibodies, and 1:1000 for secondary antibodies). The primary antibodies were incubated overnight at 4 °C. The secondary antibodies Goat anti-mouse IgG Alexa 488 (Cat. No. A11029, ThermoFisher), Donkey anti-rabbit IgG Alexa 594 (Cat. No. A21207, ThermoFisher), and DAPI were incubated in the dark for 2 h at RT. The photographs were processed by applying ImageJ software (http://imagej.nih.gov).

### Statistics

Data from qPCR and functional assays were imported into R version 4.1.2^23^ and log2-transformed prior to analysis. Statistical testing was performed with repeated measures ANOVA to identify statistically significant differences in marker gene expression and albumin/urea abundance between time points. Post-hoc testing was performed with Tukey’s test. Results were considered statistically significant where *P* < .05.

## Results

### CNN Accurately Distinguishes Between Early and Late Hepatocyte Differentiation Stages

To test our hypothesis, 1331 phase-contrast microscopy images were obtained between days 1 and 23 following the onset of differentiation of several human PSC (hPSC) lines toward hPSC-derived hepatocytes (hPSC-HEP). In accordance with the differentiation protocol, the culture medium was changed to a hepatocyte maturation medium on day 14 to direct the differentiation from hepatic progenitors into functional hepatocytes. We wanted to investigate the possibility of distinguishing between images captured before and after this time point in the protocol, based on the morphological features of the cells. Therefore, images collected during days 1-14 were labeled as early differentiation (ED, in total 693 images) and images collected during days 16-23 as late differentiation (LD, in total 638 images). No images were collected on day 15. The original images were cut and processed into a larger dataset of 6972 smaller image patches prior to analysis with CNN.

**Figure 3.**
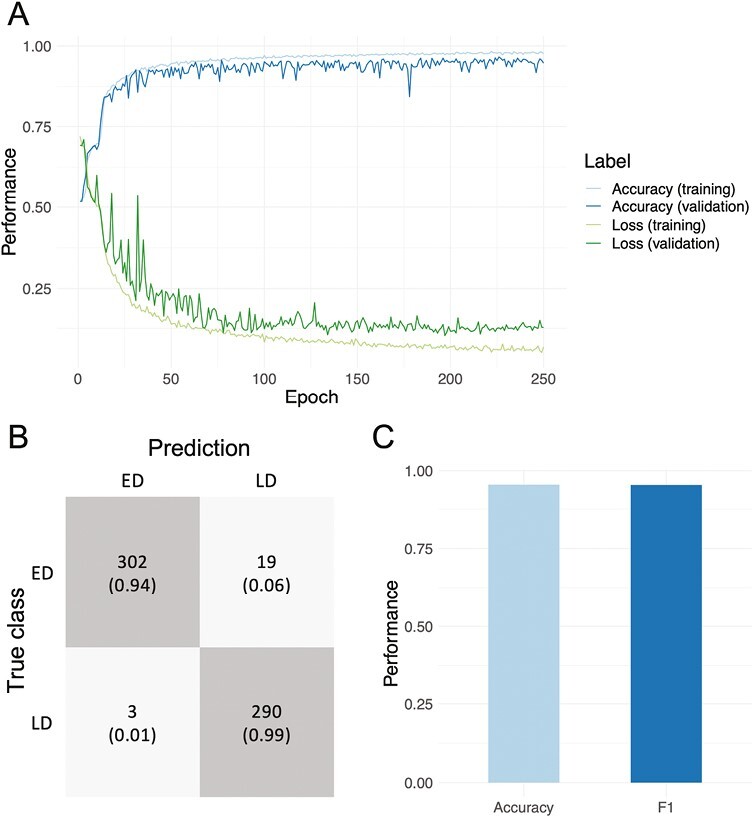
Image classification performance of the CNN. **A**. Training and validation accuracy and loss. **B**. Confusion matrix for the independent test set of 614 hiPSC images classified using the trained model. **C**. Accuracy and F1 were obtained for the independent test set. ED, early differentiation; LD, late differentiation.

A CNN architecture was then trained on 5086 images and validated on 1272 for hyperparameter tuning. The trained model was subsequently evaluated on the independent test set of 614 images to assess its predictive power. Image classification performance during training, validation, and testing is presented in Fig. 3, showing that the converged model was highly accurate. Training set accuracy converged at 0.98 after around 200 epochs and validation set accuracy at 0.95 slightly earlier, albeit with greater variation between epochs. The CNN was likewise accurate on the independent test set, where accuracy and F1 of 0.95 were achieved (Fig. 3C). This corresponded to 592 out of 614 images correctly classified as ED or LD.

**Figure 4.**
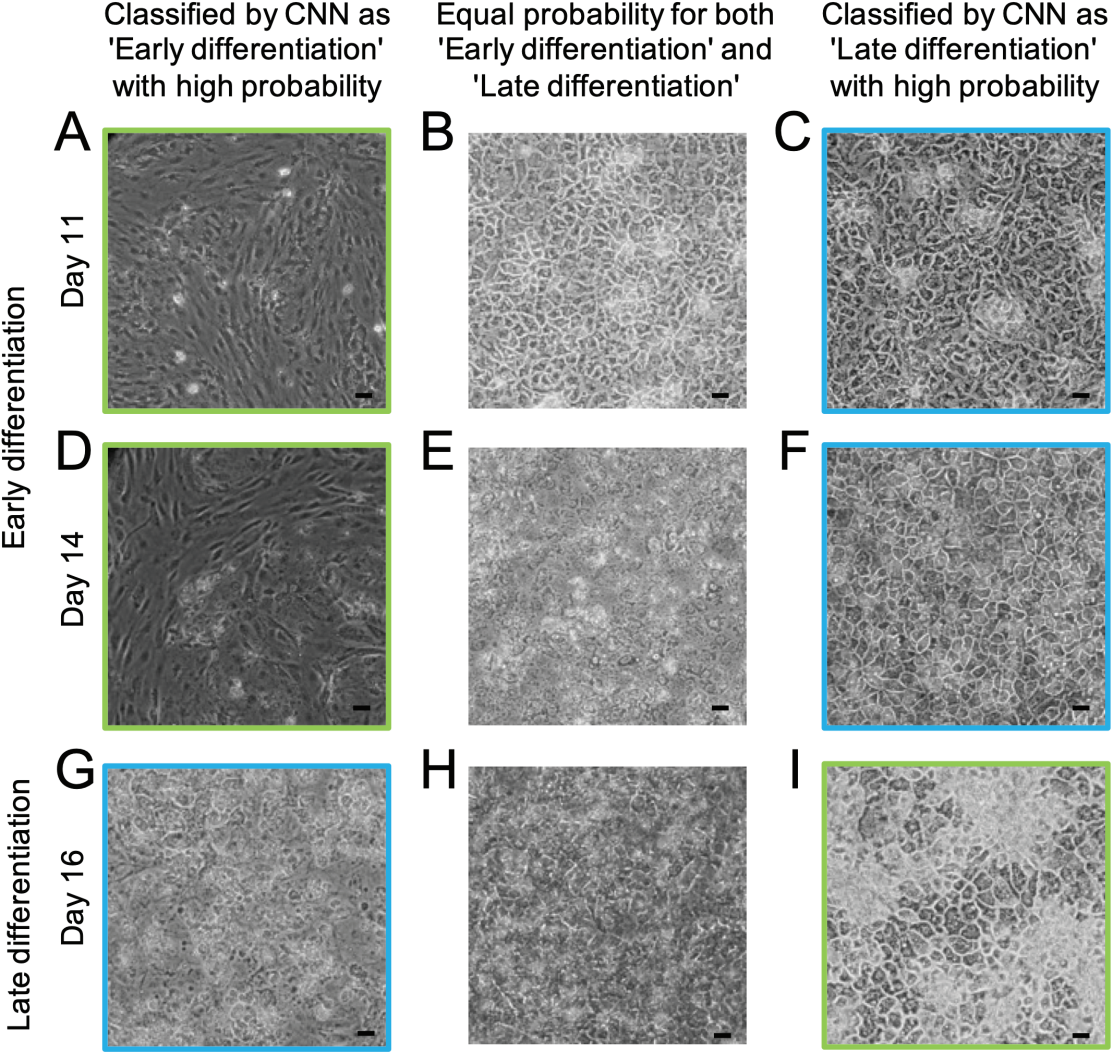
Representative images of hPSC cultures arranged according to their CNN classification. Images correctly classified with high probability for the correct class are shown in the left column for days 11 and 14 (panels A and D), and the right column for day 16 (panel I). hPSC images classified as ED and LD with approximately equal probability are presented in the middle column (panels B, E, and H). Images incorrectly classified with high probability (for the wrong class) are shown in the right column for days 11 and 14 (panels C and F) and the left column for day 16 (panel G). Scale bar: 100 µm. Magnification: 10×.

To investigate the relationship between cell culture morphology and predictions made by the CNN, we looked at cell culture images from early and late differentiation, and the reported classification probability from the CNN. This is computed by the output layer of the model and is a value between 0 and 1, where 0 corresponds to a high probability for ED and 1 to a high probability for LD. This allowed us to compare the morphology in ED and LD images that were correctly and wrongly classified, as well as where the CNN was unsure about the differentiation stage (probability close to 0.5). Representative images are shown in Fig. 4, with correctly classified in the top left and bottom right (marked by green border), wrongly classified in the top right and bottom left (marked by blue border), and images where the CNN was unsure in the middle.

A potential explanation for the incorrect classification of some images is a similar polygonal cell shape in the progenitor stage and in the hepatocyte stages. Thus, some ED pictures were incorrectly classified by the CNN model as LD, though they do not show the typical hepatocyte morphology (Fig. 4C and 4F). Similarly, the image labeled as LD, but classified by the CNN model as ED, shows cells in the early stages of differentiation where cultures were sparser than usual and the cells lost their typical morphology (Fig. 4G). Among the images that were classified with equal probability as ED and LD, the 2 top ones (Fig. 4B and 4E) are from the early stage of differentiation (days 11 and 14, respectively). One likely cause for a difficult classification of this stage is that day 14-cultures can display different morphologies depending on slight variations in cell density (slightly higher density in Fig. 4B, slightly lower in Fig. 4E). The image for day 16 (Fig. 4H) shows cells in the late stage of differentiation, but cultures at the late stage can appear slightly blurry without distinct cell borders, which makes the classification more difficult.

Stem cell differentiation is a continuous process, and it may be difficult to define an exact time point dividing the cell differentiation stage into early and late differentiation. Hence, to further investigate the ability of the CNN model to properly classify the images, the predicted differentiation stage was assessed for test set images at each day separately (Table 1). The fraction of correctly classified images was 1.00 for images taken on days 1-8 and 17-23, except for day 22, where 0.98 were correct. On days 14 and 16, which we used as a point of division between ED and LD, the fraction of correctly classified images was 0.86. According to the differentiation protocol, the differentiation process switches from the progenitor stage to hepatocyte maturation on day 14. Taking into consideration that changes in cell function and morphology are gradual, the lower fraction around day 14 is not unexpected. In addition, differences in cell morphology on day 14 caused by slight variations in cell density may contribute to the lower fraction (see explanation in the previous paragraph). However, a fraction of 0.86 implies that the CNN nonetheless can recognize morphological changes that occur within just 2 days.

**Table 1.** Classification by differentiation day on hPSC images in the independent test set.

Differentiation stage	Days of differentiation	Fraction correctly classified
**Early**	1	1.00
	2	1.00
	3	1.00
	4	1.00
	6	1.00
	7	1.00
	8	1.00
	9	0.92
	10	1.00
	11	0.97
	14	0.86
**Late**	16	0.86
	17	1.00
	18	1.00
	19	1.00
	21	1.00
	22	0.98
	23	1.00

### Experimental Validation of Cell Maturation During Late Differentiation

**Figure 5.**
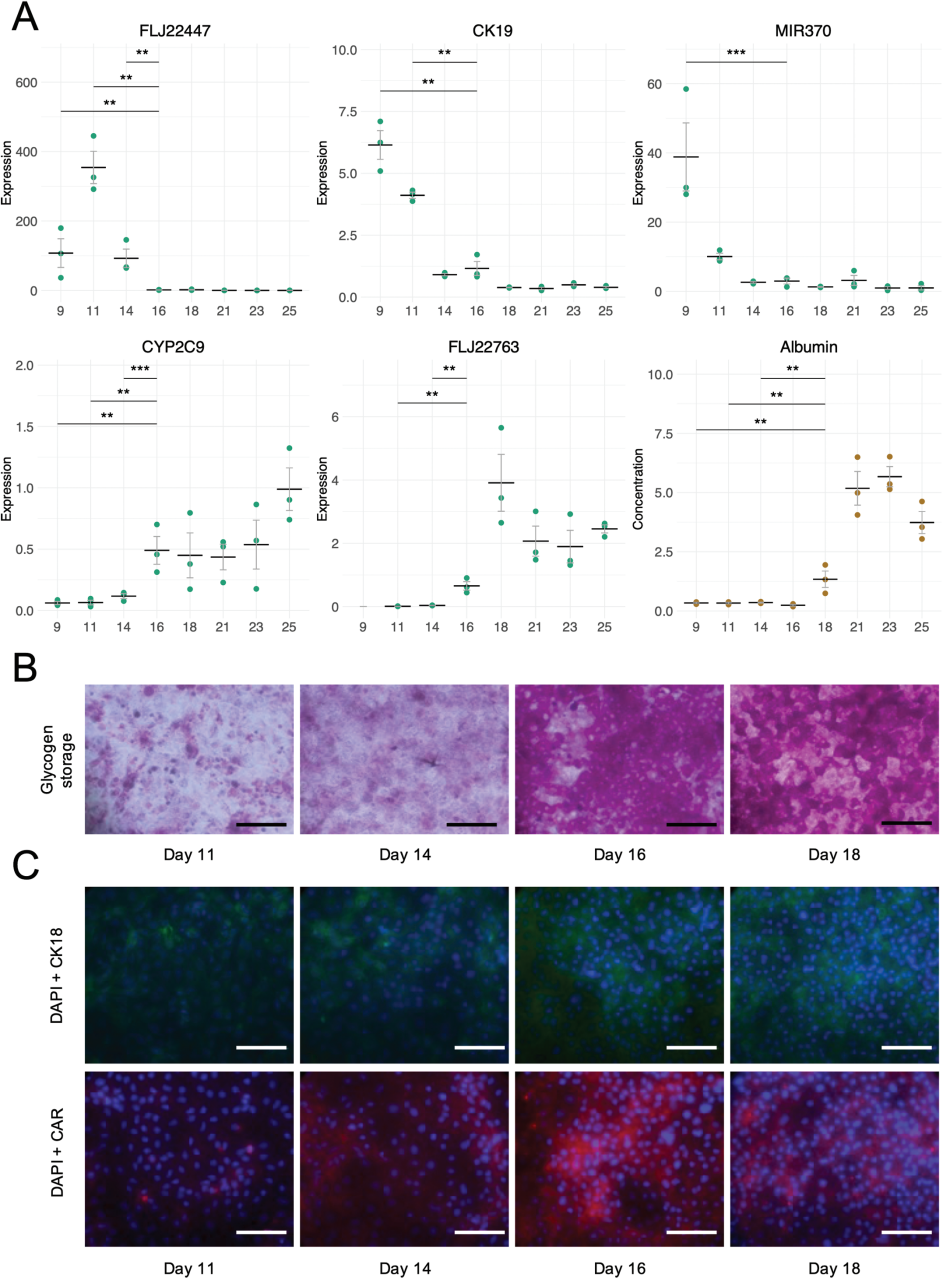
Experimental characterization of hPSCs undergoing differentiation toward hPSC-HEP. **A**: qPCR expression data for 5 markers of hepatocyte maturation and albumin secretion in μg/mg protein/24 h (*N* = 3, number of cell lines). Horizontal bar denotes the mean and error bars represent standard error. X axis shows number of days following differentiation initiation. Statistical testing was performed with repeated measures ANOVA followed the Tukey test. For clarity, only comparisons between days 9-14 and 16 (qPCR), and days 9-14 and 18 (albumin) are shown. No expression values were obtained for FLJ22763 day 9. ** *P* < .01; *** *P* < .001. **B**: Representative pictures of PAS-stained cultures visualizing glycogen storage on days 11-18. **C**: Representative pictures of immunocytochemical staining showing expression of CK18 and CAR on days 11-18. Scale bar: 100 µm. Magnification: 40×.

In practice, researchers rely on experimental assays to verify the functionality and differentiation stage of cell cultures. Four properties of cells are routinely measured to assess cell identity, namely cell morphology, gene expression, protein expression, and cell functionality. To investigate the differentiation efficiency of hPSCs toward hepatocytes, typically established marker genes and proteins, and functional assays that define the maturity of the cells are available.^24^ While the CNN was able to learn important morphological changes in microscopy images, we also wanted to experimentally characterize biological changes over the course of differentiation toward hPSC-HEP. Importantly, it was necessary to confirm that cell cultures in the late stage (corresponding to LD images) indeed have a mature hepatocyte phenotype. Therefore, experimental assays were carried out where expression of hepatic marker genes and proteins, albumin secretion, and glycogen storage were assessed at several time points. Experimental results are shown in Fig. 5.

The experimental results show clear changes in marker expression and cell function around days 14 to 16 after the onset of differentiation. One of the key functions of the liver is the metabolism of chemicals, where more than 90% of reactions are catalyzed by the cytochrome P450 family of enzymes.^25^*CYP2C9*, one of the most abundant enzymes in the adult liver, shows a significant upregulation on day 16 and expression is further elevated on day 25 (Fig. 5A). Other important liver functions include secretion of albumin and storage of glycogen. Albumin is a serum protein essential for the maintenance of oncotic pressure and is synthesized and secreted by hepatocytes.^26^ The concentration of albumin in culture media was significantly elevated on day 18 and was further increased on days 21-25 (Fig. 5A). Another important liver feature is the storage of glycogen, which serves as a reservoir of glucose for other tissues in the body, and hepatic glycogen metabolism is important for the maintenance of blood glucose levels.^27^ The experimental data shows that the amount of glycogen stored in cell cultures was strongly increased on day 16 and onward (Fig. 5B).

CK18 is a type-I intermediate filament protein highly concentrated in hepatocytes and cholangiocytes (epithelial cells of the bile duct) and comprises 5% of total liver protein.^28^ The constitutive androstane receptor (CAR), also known as NR1I3, is a member of the nuclear receptor superfamily (subfamily 1, group I, and member 3) that is almost exclusively expressed in the liver. CAR is known to interact with key signaling pathways involved in drug, energy, and bilirubin metabolism, and is an important biomarker for mature hepatocytes.^29^ The immunocytochemistry data show an increased expression of these 2 proteins in the late stages of the differentiation (days 16-18) (Fig. 5C).

These experimental results show that the hPSC cultures adopt hepatocyte features in the late stage of differentiation. We can also see that the CNN is able to classify images captured at the beginning and end of the differentiation period with a fraction correctly classified images close to 1.00 (Table 1). Thus, predictions made by the CNN based on cell culture morphology clearly reflect the underlying functional maturation of cells.

### Impact of Image Size, Dataset Size, and Data Augmentation

**Figure 6.**
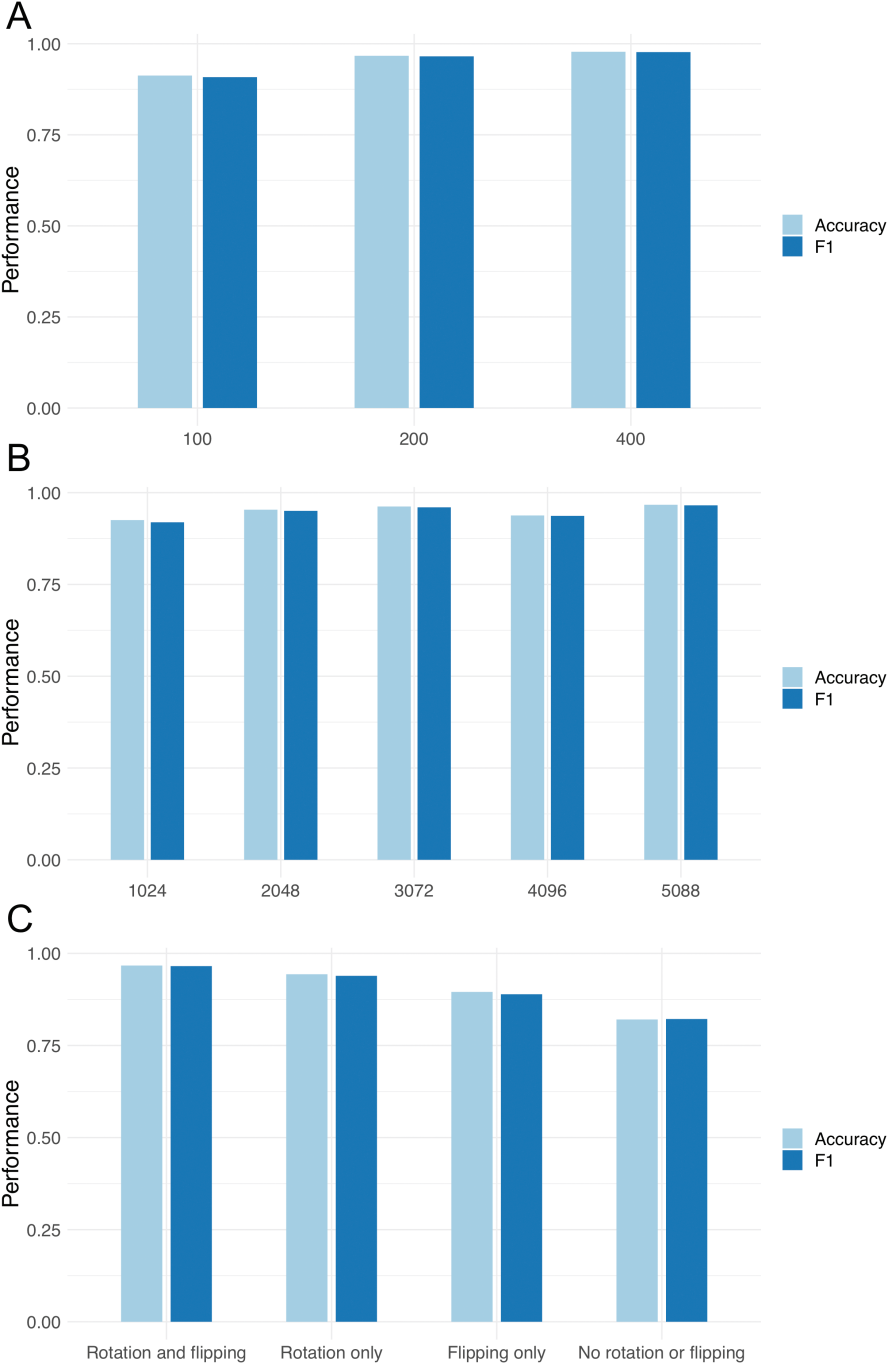
Impact of data processing on performance. Panels show impact of varying image size (**A**), dataset size (**B**), and image augmentation (**C**) on the validation accuracy and F1 of the CNN model.

To investigate the impact of image size on the training procedure, the CNN was trained with images of sizes of 400 × 400, 200 × 200, and 100 × 100 pixels. When comparing the model performance for images with resolutions 400 × 400 and 200 × 200 pixels (Fig. 6A), the lower resolution resulted in a small reduction in accuracy and F1 during the validation of the model, while the computational time required for training was reduced 3.5-fold. Further reduction of image resolution to 100 × 100 pixels resulted in a drop in accuracy and F1 from 0.96-0.97 to around 0.91. Therefore, images of size 200 × 200 were used in this study.

One of the most common problems when training the CNN model is the lack of a sufficient number of images. It is difficult to estimate how much data is required for training since it is highly dependent on how challenging the classification problem is. Nonetheless, we investigated the sensitivity to changes in sample size to provide an indication for the classification of early and late differentiated stem cells. Initially, the set of 5088 images was used for training, followed by a consecutive reduction in the number of images until 1024 images were left in the training dataset. Accuracy and F1 varied between 0.94 and 0.97 as the size of the dataset was reduced from 5088 to 2048 images (Fig. 6B). When only 1024 images were used for training the model, a reduction in accuracy and F1 to 0.92-0.93 was observed.

Data augmentation were assessed in the present study by comparing classification performance on a dataset where images were flipped and rotated, with a dataset without one/both augmentation steps. The results showed that both flipping and rotation indeed had a strong positive impact on the performance of the CNN model by mitigating overfitting (Fig. 6C). Without augmentation, accuracy on the training set was close to 1.00 (data not shown), while accuracy on the validation set was only 0.82, which indicates that the model overfitted during the training. Excluding one step of image augmentation (flipping or rotation) likewise resulted in a reduction in classification accuracy.

## Discussion

Quality control of stem cell cultures is a crucial yet laborious process. Motivated by recent successes in AI-based microscopy image analysis,^10-12^ we hypothesized that CNNs can be used to distinguish between different stages of hepatocyte differentiation, based on cell morphology in microscopy images. To evaluate this hypothesis, a CNN was trained to distinguish between images taken during early and late hepatocyte differentiation of hPSCs. Late differentiation was defined as the stage where hPSC-derived hepatic progenitor cells were differentiated further toward hPSC-derived hepatocytes (hPSC-HEP), which would correlate with morphological changes toward the functional, mature cell type. Our results showed that the CNN was highly successful at this task, achieving 0.96 accuracy on the independent test set, and close to perfect classification performance on images taken at the beginning and end of the differentiation period, when differences in typical morphology were more pronounced. The predictions made by the CNN aligned well with experimental data on gene and protein expression, and functional features such as glycogen storage, reflecting the functional maturation of cells. These results indicate that semi-automated CNN-based image analysis could serve as a complement to the extensive experimental characterization of differentiated hepatocytes. The adoption of computational methods to assess cell maturation could therefore simplify and accelerate stem cell production and research. Importantly, this would provide a less subjective method for morphological assessments than visual inspection performed by different trained researchers. It is important to note that our model has not been validated on an external dataset and that the application of CNN models to stem cell production lines would require refitting or fine-tuning model parameters to the specific lab and cell type in question. For example, our model could possibly be used for transfer learning to other hepatocyte differentiation datasets.

The results from this study showed a clear improvement in results when the image data were augmented. The assessment of data augmentation showed that the combination of rotating and flipping of images was the most successful approach, achieving a considerably higher accuracy compared to when these were not applied. Cutting the images into patches to increase dataset size also improved the performance of the model. Thus, the success of the CNN model trained in this study could therefore be largely attributed to the image augmentation and pre-processing steps. It is worth noting that we have used a fairly simple CNN model in this study, trained on approximately 1300 images. This shows that even shallow CNNs can be successfully applied to the classification of moderately sized microscopy datasets, depending on how challenging the classification problem is.

A strength of this study is the use of images from several cell lines obtained during the everyday operation of a commercial lab. The original microscopy images were not specifically prepared or selected for the purpose of training a CNN. By using images obtained during everyday laboratory work, the data will be more closely representative of the heterogeneity due to ,for example, instruments, lab personnel, reagent batches, variations in cell densities, etc. On the other hand, the images used to train, validate, and test the CNN were generated by processing the original microscopy images. If the trained CNN was applied in practice, its task would be to classify cell cultures based on new uncropped microscopy images. The data used to train a CNN should be representative of those images, so the question arises to what extent training set image processing affects performance. One way to mitigate the risk of performance reduction in practice would be to use software that pre-processes the new microscopy images in real-time, generating multiple patches per image that the CNN classifies. This would result in several differentiation stage checks for different parts of the original image. Overall culture quality could then be assessed by combining the individual stage checks.

Other CNN architectures such as VGG16^30^ and ResNet^21^ could also be investigated depending on how challenging the image classification task is. Given that our CNN achieved close to perfect accuracy, we did not explore this further. Classification performance was lower though for images captured on days near the change from progenitor medium to maturation medium. This is not unexpected, since morphological changes occur continuously during the differentiation, and no sharp distinctions can be observed at specific time points. Even when using molecular markers, it might be difficult to distinguish between cells around 14-16 days of culturing for the same reason. We do not believe this restricts the use of CNNs in future stem cell production and research though, as the cells would typically be assessed at the end of the differentiation process.

The results from this study clearly demonstrate the great potential of CNNs for addressing challenging image analysis problems related to stem cell culture characterization, where subtle differences may be of critical functional importance. However, this approach may also have great potential for quality control of stem cell products intended for regenerative medicine. In the promising area of advanced therapeutic medical products (ATMPs), the development of quality control procedures for the assessment of cell identity and cell quality is a key challenge,^31^ for which CNNs may be a viable automated approach in large-scale cell production.

## Conclusion

Characterization and quality control of differentiated stem cell cultures are laborious and time-consuming. Here we have shown that convolutional neural networks, trained to recognize the morphological features of functional cells, may serve as a complement to experimental validation and provide a means for semi-automated quality control. This has potential implications for a more efficient and objective assessment of stem cell culture quality.

## Data Availability

The data underlying this article cannot be shared publicly since the data is the property of Takara Bio Europe. For access to the data, please contact corresponding author.

## References

[1] Moen E , Bannon D, Kudo T, et al. Deep learning for cellular image analysis. Nat Methods. 2019;16(12):1233-1246. 10.1038/s41592-019-0403-1 31133758 PMC8759575

[2] Grafton F , Ho J, Ranjbarvaziri S, et al. Deep learning detects cardiotoxicity in a high-content screen with induced pluripotent stem cell-derived cardiomyocytes. Elife. 2021;10:e68714. 10.7554/eLife.68714 34338636 PMC8367386

[3] Guan B , Bhanu B, Theagarajan R, et al. Human embryonic stem cell classification: random network with autoencoded feature extractor. J Biomed Opt. 2021;26(5):52913. 10.1117/1.JBO.26.5.052913 PMC8084167 33928769

[4] Imamura K , Yada Y, Izumi Y, et al. Prediction model of amyotrophic lateral sclerosis by deep learning with patient induced pluripotent stem cells. Ann Neurol. 2021;89(6):1226-1233. 10.1002/ana.26047 33565152 PMC8247989

[5] Joy DA , Libby ARG, McDevitt TC. Deep neural net tracking of human pluripotent stem cells reveals intrinsic behaviors directing morphogenesis. Stem Cell Rep. 2021;16(5):1317-1330. 10.1016/j.stemcr.2021.04.008 PMC8185472 33979602

[6] Maddah M , Mandegar MA, Dame K, et al. Quantifying drug-induced structural toxicity in hepatocytes and cardiomyocytes derived from hiPSCs using a deep learning method. J Pharmacol Toxicol Methods. 2020;105:106895. 10.1016/j.vascn.2020.106895 32629158

[7] Zhang Z , Leong KW, Van Vliet K, Barbastathis G, Ravasio A. Deep learning for label-free nuclei detection from implicit phase information of mesenchymal stem cells. Biomed Opt Express. 2021;12(3):1683-1706. 10.1364/BOE.420266 33796381 PMC7984805

[8] LeCun Y , Bengio Y, Hinton G. Deep learning. Nature. 2015;521(7553):436-444. 10.1038/nature14539 26017442

[9] Waisman A , La Greca A, Möbbs AM, et al. Deep learning neural networks highly predict very early onset of pluripotent stem cell differentiation. Stem Cell Rep. 2019;12(4):845-859. 10.1016/j.stemcr.2019.02.004 PMC6449871 30880077

[10] Liu Y , Zhang Y, Cui J. Recognized trophoblast-like cells conversion from human embryonic stem cells by BMP4 based on convolutional neural network. Reprod Toxicol. 2021;99:39-47. 10.1016/j.reprotox.2020.11.006 33249234

[11] Zhu Y , Huang R, Wu Z, et al. Deep learning-based predictive identification of neural stem cell differentiation. Nat Commun. 2021;12:1-13. 10.1038/s41467-021-22758-0 33972525 PMC8110743

[12] Lan Y , Huang N, Fu Y, et al. Morphology-based deep learning approach for predicting osteogenic differentiation. Front Bioeng Biotechnol. 2021;9:1521. 10.3389/fbioe.2021.802794 PMC8830423 35155409

[13] Coronnello C , Francipane MG. Moving towards induced pluripotent stem cell-based therapies with artificial intelligence and machine learning. Stem Cell Rev Rep. 2021;18:559-569. 10.1007/s12015-021-10302-y 34843066 PMC8930923

[14] Orita K , Sawada K, Koyama R, Ikegaya Y. Deep learning-based quality control of cultured human-induced pluripotent stem cell-derived cardiomyocytes. J Pharmacol Sci. 2019;140(4):313-316. 10.1016/j.jphs.2019.04.008 31113731

[15] Hirose T , Kotoku J, Toki F, Nishimura EK, Nanba D. Label-free quality control and identification of human keratinocyte stem cells by deep learning-based automated cell tracking. Stem Cells. 2021;39(8):1091-1100. 10.1002/stem.3371 33783921 PMC8359832

[16] Piotrowski T , Rippel O, Elanzew A, et al. Deep-learning-based multi-class segmentation for automated, non-invasive routine assessment of human pluripotent stem cell culture status. Comput Biol Med. 2021;129:104172. 10.1016/j.compbiomed.2020.104172 33352307

[17] Ghosheh N , Küppers-Munther B, Asplund A, et al. Human pluripotent stem cell-derived hepatocytes show higher transcriptional correlation with adult liver tissue than with fetal liver tissue. ACS Omega. 2020;5(10):4816-4827. 10.1021/acsomega.9b03514 32201767 PMC7081255

[18] Holmgren G , Ulfenborg B, Asplund A, et al. Characterization of human induced pluripotent stem cell-derived hepatocytes with mature features and potential for modeling metabolic diseases. Int J Mol Sci. 2020;21(2):21. 10.3390/ijms21020469 PMC7014160 31940797

[19] Asplund A , Pradip A, van Giezen M, et al. One standardized differentiation procedure robustly generates homogenous hepatocyte cultures displaying metabolic diversity from a large panel of human pluripotent stem cells. Stem Cell Rev Rep. 2016;12:90-104. 10.1007/s12015-015-9621-9 26385115

[20] Gulli A. Pal S. Deep Learning with Keras. Packt Publishing Ltd; 2017.

[21] He K , Zhang X, Ren S, Sun J. Deep residual learning for image recognition. Proc IEEE Conf Comput Vis Pattern Recognit. 2016:770-778. 10.1109/CVPR.2016.90

[22] Kingma DP , Ba J. 2014. Adam: a method for stochastic optimization. *ArXiv*, 10.48550/arXiv.1412.6980.

[23] R Core Team. R: A Language and Environment for Statistical Computing, Vienna, Austria; 2021.

[24] Zhao D , Chen S, Duo S, et al. Promotion of the efficient metabolic maturation of human pluripotent stem cell-derived hepatocytes by correcting specification defects. Cell Res. 2013;23(1):157-161. 10.1038/cr.2012.144 23070301 PMC3541654

[25] Rendic S , Guengerich FP. Survey of human oxidoreductases and cytochrome P450 enzymes involved in the metabolism of xenobiotic and natural chemicals. Chem Res Toxicol. 2015;28(1):38-42. 10.1021/tx500444e 25485457 PMC4303333

[26] Buyl K. , De Kock J., Bolleyn J., Rogiers V, Vanhaecke T. Measurement of albumin secretion as functionality test in primary hepatocyte cultures. In Protocols in In Vitro Hepatocyte Research, Springer; 2015: 303–308. 10.1007/978-1-4939-2074-7_22 26272152

[27] Bollen M , Keppens S, Stalmans W. Specific features of glycogen metabolism in the liver. Biochem J. 1998;336(1):19-31. 10.1042/bj3360019 9806880 PMC1219837

[28] Uhlén M , Fagerberg L, Hallström BM, et al. Proteomics. Tissue-based map of the human proteome. Science. 2015;347(6220):1260419. 10.1126/science.1260419 25613900

[29] Bae SDW , Nguyen R, Qiao L, George J. Role of the constitutive androstane receptor (CAR) in human liver cancer. Biochim Biophys Acta (BBA)—Rev Cancer. 2021;1875(2):188516. 10.1016/j.bbcan.2021.188516. 33529650

[30] Simonyan, K, Zisserman A. 2014. Very deep convolutional networks for large-scale image recognition. *ArXiv*, 10.48550/arXiv.1409.1556.

[31] Beheshtizadeh N , Gharibshahian M, Pazhouhnia Z, et al. Commercialization and regulation of regenerative medicine products: Promises, advances and challenges. Biomed Pharmacother. 2022;153:113431. 10.1016/j.biopha.2022.113431 36076549

